# 再生障碍性贫血诊断与治疗中国指南（2022年版）解读

**DOI:** 10.3760/cma.j.issn.0253-2727.2023.03.003

**Published:** 2023-03

**Authors:** 蓉 付, 婷 王

**Affiliations:** 天津医科大学总医院血液科，天津 300052 Department of Hematology, Tianjin Medical University General Hospital, Tianjin 300052, China

再生障碍性贫血（aplastic anemia, AA）是血液系统重症，以外周全血细胞减少和骨髓造血功能衰竭为主要表现。从1888年AA被命名至今已百余年历史，其发病的核心机制已被归结为T细胞功能亢进介导的免疫性骨髓衰竭（BMF）。AA的治疗经历了糖皮质激素、雄激素、造血干细胞移植、抗人胸腺T细胞免疫球蛋白（ATG）、ATG联合环孢素A（CsA）等诸多阶段，近期全球多个临床研究均显示TPO受体激动剂（TPO-RA）可显著提升AA免疫抑制治疗（IST）的疗效，特别是对重型再生障碍性贫血（SAA）尤为突出。基于AA研究新进展及越来越多的高级别循证医学证据，中华医学会血液学分会红细胞疾病（贫血）学组在《2017版AA诊断与治疗中国专家共识》[1]的基础上更新了部分内容，并修订为《再生障碍性贫血诊断与治疗中国指南（2022年版）》（简称新版指南）[2]，以便更好地推动我国临床医师对AA诊疗的规范化，进一步提高我国AA患者的疗效。现对修订后的新版指南解读如下，以供参考。

一、AA的诊断

1. 免疫学发病机制：强调免疫学发病机制为AA发病的核心机制。T淋巴细胞的异常活化为主导作用，多种免疫细胞亚群参与其中：辅助性T细胞亚群Th1/Th2分化偏移[3]，Th1数量增多、功能增强，Th2比例功能不足；调节性T细胞（Treg）及NK细胞免疫负调控作用不足[4]–[6]；Th17[7]数量增多、功能增强在AA发病炎症因子风暴中起到推波助澜的作用；树突状细胞（DC），尤其是髓样DC（mDC）是最主要的抗原提呈细胞，在AA患者中其抗原提呈作用增强[8]；近期还发现巨噬细胞也参与AA发病，小鼠实验证实巨噬细胞是造成AA免疫微环境损伤的重要原因[9]，本指南增加了巨噬细胞参与AA发病的部分内容。

在新版指南的AA实验室检测项目中，除了血常规、网织红细胞，不同水平面骨髓穿刺、骨髓活检，生化、免疫、造血原料、病毒等常规检查项目之外，还推荐了流式细胞术进行免疫细胞亚群的检查，包括T细胞亚群（如CD4^+^、CD8^+^、Th1、Th2、Treg等）、NK细胞亚群、细胞因子（如IFN-γ、IL-4、IL-10等），上述检查不仅是AA诊断的有力佐证，也是后期评估AA治疗后免疫恢复的重要监测指标，建议AA确诊时务必完善上述检查。

2. 细胞遗传学异常：细胞遗传学异常包括体细胞异常和胚系异常，前者又包括基因突变导致的克隆造血（CH）及染色体异常。AA患者体细胞遗传学异常的发生率明显高于普通人群，与年龄呈正相关。AA患者发病时外周血细胞端粒明显缩短，与疾病的严重程度和IST疗效相关[10]–[11]。AA最常见的体细胞突变基因为BCOR/BCORL1、PIGA、DNMT3A和ASXL1[12]；其中BCOR/BCORL1、PIGA与IST治疗反应相关；DNMT3A和ASXL1为普通人群中常见的CH，与年龄及免疫炎症压力密切相关，提示AA预后不良。除DNMT3A、ASXL1外，RUNX1、TP53、CSMD2基因突变也预示AA预后不良，向骨髓增生异常综合征/急性髓系白血病（MDS/AML）高风险转化[13]。由于AA是排除性诊断，因此对于携带MDS/AML高风险转化基因的患者或携带明确染色体核型异常的患者诊断AA应极为慎重。2022年WHO对髓系肿瘤分类标准中指出：低增生性MDS（MDS-h）发病机制同样为寡克隆性CD8^+^ T细胞介导的对造血干细胞及造血前体细胞的免疫攻击，与AA发病机制高度重合，MDS-h对ATG等免疫抑制治疗也有效，但疾病的本质为恶性病，需与AA严格鉴别。对于胚系基因突变，GATA2、DDX41、范可尼贫血基因、端粒复合体基因突变等，也可表现为骨髓衰竭，需仔细与AA甄别[14]。

在新版指南中强调了细胞遗传学检查的重要性，不仅包括染色体核型分析、特殊位点的荧光原位杂交（FISH）检测，更是建议有条件的医院进行二代测序（NGS）、端粒酶活性及端粒长度检测；同时新版指南也将需要与AA鉴别的先天性BMF明确列出，并建议在与AA鉴别诊断时，进行先天性BMF相关基因突变及克隆造血分子标志的检查；力求实现各级医院对AA诊断的统一和精准。

对其他需要与AA鉴别的原发或继发性BMF新版指南未做大的变更。

3. AA严重程度的确定：新版指南对于SAA的诊断标准，仍遵循Camitta标准。根据是否依赖血制品输注，将非重型AA（NSAA）分为输血依赖型（TD-NSAA）和非输血依赖型（NTD-NSAA）。由于NTD-NSAA预后相对较好，而TD-NSAA高风险向SAA转化，如按照NTD-NSAA治疗可能因免疫抑制治疗不足等原因导致疗效欠佳，应尽早按照SAA的治疗策略给予治疗。因此区分TD-NSAA和NTD-NSAA是十分必要的。

本指南明确了成分输血的指征：HGB≤60 g/L；PLT≤10×10^9^/L或PLT≤20×10^9^/L伴有明显出血倾向。平均每8周至少1次成分输血且输血依赖持续时间≥4个月定义为TD-NSAA。该部分参考了《成人重型和输血依赖的非重型再生障碍性贫血中西医结合诊疗专家共识》[15]部分内容，以便各中心对TD-NSAA诊断的同质化。

二、AA的治疗

1. AA整体治疗原则：多个回顾性研究均发现SAA越早治疗预后越好，尤其是确诊30 d之内启动治疗的SAA患者，预后明显优于30 d之后启动治疗者[16]。因此新版指南特别强调了SAA尽早启动治疗的必要性。HLA相合同胞供者造血干细胞移植（MSD-HSCT）仍然是SAA适合移植患者的首选治疗方案。随着我国造血干细胞移植领域的发展，本指南将一线移植SAA患者的年龄从2017版的≤35岁放宽至≤40岁。对那些一线不适合MSD-HSCT的SAA患者，指南建议将IST（ATG+CsA）联合TPO-RA的治疗方案作为基石，其他促造血治疗在上述三药方案的基础上酌情增加。新版指南还明确指出对TD-NSAA及NTD-NSAA应采用不同的治疗策略：对于TD-NSAA，参照SAA进行治疗；对于NTD-NSAA，可采用CsA联合TPO-RA为基础的治疗方案。

2. IST联合TPO-RA方案：

（1）疗效获益：TPO-RA是近30年来SAA治疗的最大亮点。欧洲骨髓移植工作组数据显示：相比较单用IST治疗组，IST联合艾曲泊帕组将12个月治疗完全缓解（CR）率由33％提升至52％；首次治疗反应中位时间由8.8个月缩短至3个月；从部分缓解（PR）到CR所需中位时间由5.1个月缩短至2.7个月；最佳反应的中位时间由8.9个月缩短至3.9个月；24个月无事件生存率由34％提升至46％。对治疗有效患者，脱离输注红细胞的时间由140 d缩短至51 d。除年龄和疾病严重程度之外，是否联合应用TPO-RA是SAA 6个月治疗反应的独立影响因素[17]。美国国立卫生研究院（NIH）结果也显示SAA患者6个月治疗总反应率（ORR）在IST联合艾曲泊帕组高达94％，CR率高达58％，远高于单用IST组的ORR 66％和CR率17％[18]–[19]。我国研究也显示无论初治还是复发难治的SAA，艾曲泊帕都显示出良好的疗效[20]–[21]。根据上述研究结果，新版指南提出将IST联合TPO-RA的方案作为一线不适合MSD-HSCT的SAA患者的首选治疗方案和基石方案。

（2）应用时机及疗程：在方案使用上NIH[19]的研究显示：组3（ATG治疗第1天同时应用艾曲泊帕至6个月组）6个月ORR及CR率均明显优于组2（ATG后14 d到6个月加用艾曲泊帕组）和组1（ATG后14 d到3个月加用艾曲泊帕组），因此本指南推荐TPO-RA与ATG第一天同时应用以获得最佳疗效。由于艾曲泊帕在亚洲人群的清除率较欧洲人群低33％～52％[22]，因此艾曲泊帕治疗SAA在我国推荐的起始剂量为75 mg，最大剂量为150 mg。NIH[18]研究还显示，IST联合艾曲泊帕治疗SAA患者复发多发生在3个时间节点：①6个月停用艾曲泊帕后；②6个月停药或减量CsA后；③24个月停用CsA后。艾曲泊帕停药后复发可能与SAA患者骨髓储备功能不足有关，比如老年人或6个月未达CR者更容易出现停用艾曲泊帕后的复发，其中85％表现为单纯血小板减少，因此建议艾曲泊帕逐渐减量后停药。对CsA维持治疗的疗程，长久以来存在争议，英国AA指南提出[23]：CsA快速减量存在很大的复发风险，建议在达到最佳反应后至少维持治疗12 个月，然后再开始缓慢减量，如3个月减25 mg。NIH研究显示：如果CsA仅应用6个月即停药，可导致56％患者复发，当CsA维持治疗时间延长至24个月时，复发率下降至约30％，治疗有效率提升了17％。国内研究也显示ATG治疗后CsA应足量使用6个月或疗效达平台期后继续维持用药12～24个月后逐渐停药，此法可明显减低SAA复发概率[24]。因此新版指南一方面明确了成人CsA目标血药浓度（C0谷浓度）尽量达到150～250 µg/L[25]，另一方面确定了CsA的使用疗程，即足量应用6个月或疗效达平台期后建议持续用药12～24个月后停药。

（3）TPO-RA的选择及转换：目前国内能获得的TPO-RA包括海曲泊帕、艾曲泊帕、阿伐曲泊帕、罗米司亭。其中海曲泊帕在中国获批了成人难治SAA适应证，剂量为7.5～15 mg/d；艾曲泊帕是目前全球机制研究最为透彻、应用随访时间最长的TPO-RA，在美国获批了初治及复发AA的适应证，亚洲人群推荐剂量为75～150 mg/d；阿伐曲泊帕在肝功能异常的SAA患者中有一定优势，但尚未获批AA适应证。若某一种TPO-RA疗效不佳时，可进行药物之间的相互转化，目前有确切转换后疗效数据的临床研究为艾曲泊帕治疗无效SAA患者换用罗米司亭（起始剂量每周20 µg/kg）治疗，3个月治疗有效率可达70％[26]。其他药物相互转换的临床试验仍在进行之中。

（4）疗效影响因素：在2017版IST疗效预测因素的基础上，新版指南增加BCOR/BCORL1突变为IST疗效良好的预测因素[27]。对于哪一部分SAA患者能够从IST联合TPO-RA的三药方案中明显获益，目前研究显示：网织红细胞绝对值（ARC）>30×10^9^/L的SAA患者加与不加艾曲泊帕的治疗ORR分别为89％和90％，差异无统计学意义；对于ARC在（10～30）×10^9^/L的SAA患者疗效提升最为显著，ORR由单纯IST治疗组的60％提升至91％；对于ARC<10×10^9^/L的SAA患者，IST联合艾曲泊帕虽然未能显著提升治疗ORR（63％对46％，*P*＝0.062），但CR率明显增高（27％对8％）。因此对于网织红细胞较低的SAA患者艾曲泊帕的加入优势更为显著[28]。此外年龄和骨髓造血残留（淋巴细胞绝对值与ARC）在TPO-RA时代仍然是预测IST疗效最有效的指标[29]。

3. 造血干细胞移植治疗：新版指南参考了《中国异基因造血干细胞移植治疗血液系统疾病指南2022》[30]，该指南提出MSD-HSCT是新诊断≤50岁SAA的首选治疗选择，如果无MSD，单倍体移植和10/10全合非血缘移植均可。尽管有研究显示基于阿仑单抗的allo-HSCT治疗年龄≥50岁或<50岁的SAA患者，其1年的无移植物抗宿主病（GVHD）无复发生存（GRFS）率相似（84％对94％），急慢性GVHD的发生率也相似（5％对4％，18％对14％），1年移植相关死亡率两组间差异无统计学意义（14％对5.4％）[31]–[32]。甚至在某些研究中，年龄≥50岁的SAA患者进行allo-HSCT，多因素分析显示年龄与移植后生存无显著相关性，仅与Ⅱ～Ⅳ级GVHD相关[33]。我国部分有经验的移植中心也将allo-HSCT一线治疗SAA年龄放宽至≤50岁，二线挽救治疗年龄放宽至50～60岁，并取得了良好的疗效。但多数研究仍然认同SAA患者年龄与移植后长期生存密切相关，MSD-HSCT 10年OS率在1～20岁组高达86％，21～40岁组可达76％，而≥40岁组仅为55％，且无论预处理方案或支持治疗如何改善，5年OS率在40岁以上人群组均无显著改善（2001−2009年为61％，2020−2015年为58％），其中40～49岁组为67％，50～59岁组为58％，>60岁组仅为45％[34]–[37]。综上，新版指南仍延续将40岁作为首选MSD-HSCT或IST联合艾曲泊帕的年龄界限。

此外，新版指南提出对于HLA相合无关供者移植（MUD-HSCT），除了用于IST失败后的二线治疗，在一些特殊条件下，也可以提至一线使用。研究显示对于儿童及青少年SAA患者，MUD-HSCT与MSD-HSCT具有相似的疗效，2年OS率分别为96％和91％，因此对于儿童SAA患者部分指南已将MUD-HSCT推向一线。尤其对于那些情况紧急，如合并危及生命的感染、细胞遗传学异常、且能迅速找到合适供者的年轻患者。基于此，本指南将年龄<20岁的年轻且情况紧急的患者划分出来，可考虑提前使用MUD-HSCT[38]–[39]。除上述变更外，新版指南还对预处理方案和GVHD的预防策略以及疗效和预后等方面提出了建议。

4. 特殊情况AA的处理：新版指南对肝炎相关性AA（HAAA）的处理提出新的建议，基于TPO-RA在AA中的疗效，我国多个临床中心将阿伐曲泊帕应用于AA并取得了值得肯定的疗效[40]–[41]，尽管目前阿伐曲泊帕在国内并未获批AA适应证，但指南推荐对于HAAA或AA伴有肝功能异常者，可尝试使用阿伐曲泊帕治疗。

5. AA的疗效标准：新版指南综合吸纳了多个AA指南、共识、临床试验以及AA疗效评价体系，最终确立了新的SAA及NSAA的疗效评价标准，以期实现国内外评价体系的可比性和一致性。对于NSAA的疗效评价标准，上一版共识并未明确写出，本版指南将NSAA治疗后CR、PR及无效（NR）标准逐一写明，供临床医师参考使用。
